# Prevalence of elevated hepatic transaminases among Jordanian patients with type 2 diabetes mellitus

**DOI:** 10.4103/0256-4947.59369

**Published:** 2010

**Authors:** Layla Judi, Ala Toukan, Yousef Khader, Kamel Ajlouni, M. Amer Khatib

**Affiliations:** a From the National Center for Diabetes Endocrinology and Genetics, Amman, Jordan; b From the Liver Department, Jordan University Hospital, Amman, Jordan; c From the Department of Public Health, Community Medicine and Family Medicine, Jordan University of Science and Technology, Irbid, Jordan

## Abstract

**BACKGROUND AND OBJECTIVES:**

Since the extent of elevation of liver transaminases in type 2 diabetics in Jordan and most of the Middle East is unknown, we estimated the prevalence of elevated liver transaminase levels among patients with type 2 diabetes and determined associated risk factors.

**METHODS:**

This study was performed on 1014 consecutive type 2 diabetic outpatients who attended the National Center for Diabetes, Endocrinology and Genetics in Amman, Jordan. The patients' age ranged between 26-85 years with a mean age of 56.8 (+9.8). Three- hundred and fifty three (54.5%) were males with a median age of 58 years (ranging between 26-82 years), and four hundred and sixty one (45.5%) were females with a median age of 57 years (ranging between 28-85 years). Body mass index, waist circumference, lipid profile, and hepatic transaminase levels were recorded. Ultraonography was performed in those with elevated alanine transaminase levels.

**RESULTS:**

Overall, the prevalence of elevated alanine transaminase (ALT) level was 10.4% (n=105) with the gender-wise prevalence being 12.8% (n=71) in men and 7.4% (n=34) in women. The prevalence of elevated aspartate transaminase (AST) levels was 5.4% (n=56) with the gender-wise prevalence being 5.6% (n=31) in men and 5.4% (n=25) in women.. Only 4.5% (n=44) showed elevated levels of both ALT and AST. Male gender (OR=2.35, CI:1.5-3.8) and high waist circumference (OR=1.9, CI:1.2-3.2) were associated with increased risk of elevated ALT levels. Younger patients had a higher tendency to have elevated ALT compared to those over 65 years (OR=12.4 for patients aged 25-45years, and OR=5.8 for those who were 45-65 years old). Non-insulin use was associated with a high odds ratio for elevated ALT levels (OR=1.7, CI: 1.1-2.9).

**CONCLUSIONS:**

Elevated ALT and AST levels are found in 10.4% and 5.4% of our type 2 diabetic patients respectively. Male gender, younger age, higher waist circumference; as an indicator of central obesity, as well as non insulin use are independent predictors of elevated liver transaminase levels.

Diabetes mellitus is known to be associated with a number of liver disorders, including isolated elevation of liver enzyme levels, nonalcoholic fatty liver disease (NAFLD), and other chronic liver disorders like hepatitis C infection (HCV), cirrhosis and hepatocellular carcinoma.^1^–^3^ NAFLD is a common indolently progressive liver disease and may range from simple steatosis of the liver, through the more severe nonalcoholic steatohepatitis (NASH) to advanced fibrosis and cirrhosis.^4^ NAFLD resembles alcohol induced liver disease, but develops in subjects who are not heavy alcohol drinkers, and who have negative tests for viral, autoimmune and metabolic liver disease. NAFLD has been related to insulin resistance and obesity, suggesting that it may represent yet another component of the metabolic syndrome.^5^,^6^

It is thought that decreased insulin sensitivity, a cornerstone in the pathogenesis of type 2 diabetes (T2D), activates lipolysis leading to increased plasma levels of non-esterified fatty acids. A resultant chronic increase in fatty acid flux from the fat stores (mainly abdominal) to non-adipose tissues such as the liver, contributes to the hepatic steatosis.^7^,^8^ Abnormal liver function test results are more common in diabetes mellitus than in the non diabetic population as well as in patients with T2D than in those with type 1diabetes.^9^,^10^

Elevated activities of the two serum transaminases; alanine transaminase (ALT) and aspartate transaminase (AST) maybe associated with liver disease. Elevation of the levels of any of the two enzymes has been found in 7.9% of the general population,^11^ whereas the prevalence of high ALT levels may reach 20% in diabetics^12^. Elevation of these enzymes is strongly related to obesity, diabetes and dyslipidemia, and their measurement may act as a surrogate marker of NAFLD presence.^2^,^4^ Of the two enzymes, ALT appears to have a role in gluconeogenesis,^13^ and seems to be more related to liver fat accumulation than AST.^14^ Some authors have suggested that minor elevation of this enzyme's level may be a good predictor of mortality from liver disease.^15^ A study of the association of serum ALT activity and ten-years' risk of cardiovascular disease (CVD)in participants of the Third National Health and Nutrition Examination Survey (NHANES- III) had reported that those with elevated ALT levels had a higher calculated CVD risk than those with normal ALT activity, if viral hepatitis or excessive alcohol consumption were excluded.^16^

The activity of ALT in the hepatocytes is 7000 fold greater than in the serum,^17^ and this abundance is the reason for using it as a marker for NAFLD in many epidemiological studies.^18^ Clark et al. proposed that elevated AST or ALT levels are predictive of the presence of NAFLD if two basic criteria are met: 1) exclusion of alternative chronic liver diseases, e.g. alcoholic liver disease, hepatitis B or C infection, and hemochromatosis; and 2) presence of features of the metabolic syndrome.

The magnitude of elevated levels of liver transaminases in the Jordanian population is currently unknown, and few studies in the Middle East have addressed this issue.^19^–^21^ This study was conducted to estimate the prevalence of elevated liver transaminase levels among patients with type 2 diabetes and to determine the associated factors.

## METHODS

All patients aged ≥25 with type 2 diabetes who attended the National Center for Diabetes, Endocrinology and Genetics (NCDEG) Amman, Jordan in the period between August-December 2007 were consecutively invited to participate in the study. A total of 1014 patients agreed to participate with a response rate of 98.4%. Selecting consecutive patients over a period of five months in the year and high response rate make the sample more representative of the population of patients attending NCDEG. The sample size of 1014 patients exceeded the minimum required sample of 682 that was calculated at a level of significance of 0.05 and power of 80% assuming that the expected prevalence is 10% and null hypothesis value is 7%. The sample size was calculated using EpiCalc (version 1.02).

Patients were interviewed during their periodic visit to the center, and their data were recorded in a standardized data sheet. Data collected included age, gender, marital status, level of education and co-morbid conditions like hypertension and dyslipidemia. Patients were asked for history of alcohol consumption and the medications used, mainly hepatotoxic drugs as steroids, antiepileptics, amiodarone and antineoplastic drugs. The study was approved by the Ethics Committee in NCDEG and informed consent obtained from all participants.

### Anthropometric measurements

Blood pressure readings were taken with patients in the sitting position. Patients were labeled as hypertensive if they were already on antihypertensive medication, or were found to have systolic blood pressure equal or higher than130 mmHg and/or diastolic blood pressure equal or higher than 80 mmHg on two occasions separated by at least one week.^22^ Body weight was taken while the patients barefooted and in light clothing, using a Detecto® scale with accuracy of ±100 g. Standing height was measured without shoes to the nearest cm using a stadiometer with the shoulders in a relaxed position and the arms hanging freely Body mass index. (BMI) (kg/m^2^) was calculated as the ratio of weight (kilograms) to the square of height (meters). Patients' BMI was classified according to WHO classification,^23^ as being normal (BMI; 18.5 to 24.9 kg/m^2^), overweight (BMI; 25 to 29.9 kg/m^2^) or obese (BMI>30 kg/m^2^). Waist circumference was measured in a standing position at the level of the umbilicus, using non stretchable tailor measuring tape and results were analyzed as categorical variable with the circumference considered abnormal if it exceeded 88cm in women, and 102cm in men.^24^

### Biochemical tests

Morning samples of venous blood were collected from patients after fasting for at least 14 hours and tested for glucose, hemoglobin A1C (HbA1c) [Using High Performance Liquid Chromatography method, supplied by Bio-Rad], for the liver enzymes; ALT and AST [Using COBAS INTEGRA systems to measure the quantitative determination of the catalystic enzyme activity, supplied by Roche Diagnostics], and for lipid profile including total cholesterol (TC), high density lipoprotein (HDL), triglycerides (TG), and low density lipoprotein (LDL) [By using the Enzymatic Colometric method by COBAS INTEGRA systems supplied by Roche Diagnostics]. Elevated ALT and AST levels were defined as enzyme activity >40 U/L, and >38 U/L respectively according to the clinical assay adopted by the center's laboratory. The degree of ALT elevation was categorized to mild (one to two times the upper limit of normal), moderate (>two to three times the upper limit of normal) and marked (more than three times the normal). To overcome the day to day variation, liver enzyme measurements were repeated after one month in patients who showed high transaminase values.^25^ As all the patients visited the clinic regularly, repeated serum transaminases measurements were available for each at least two times per year. Of those patients with normal ALT levels, none were found to have had any previous increases in the enzyme levels. The periodic liver function tests performed for all the patients in the Center also helped to detect and monitor any future changes that might be related to the use of drugs which are potentially hepatotoxic like statins.

Serological tests for the hepatitis B surface antigen (HBsAg) and HCV were requested for patients who were not initially known to be seropositive for viral hepatitis. Viral hepatitis screen was done only for patients who showed elevation in transaminase readings.HBsAg and HCV were measured using micro particle enzyme immunoassay (MEIA) technology.

### Radiological examination

NAFLD was diagnosed by an ultrasound scan of the liver performed by an experienced radiologist who was blinded to the laboratory values, by using Philips HDI 5000 Sono CT with a probe array (C5-2). A fatty liver was diagnosed if there was diffuse increase in liver echogenicity as compared with that of the kidneys.^26^ Only patients with abnormally high ALT values had undergone liver scans, after excluding causes that might elevate the transaminase levels.

### Criteria for exclusion

Subjects who were excluded from the study, were those who reported alcohol consumption, patients who are known to be seropositive for HBsAg or HCV antibodies, and those who had a clinical or biochemical evidence of autoimmune hepatitis, primary liver cirrhosis, hemochromatosis or Wilson disease.

### Statistical analysis

Data analysis was performed using the Statistical Package for Social Sciences (SPSS), version 11.5. Continuous variables were summarized as means (SD), and categorical variables as frequencies and percentages. The chi-square (χ^2^) test was used to determine the association of elevated ALT and AST levels with different variables. Multivariate analysis using logistic regression analysis was used to determine factors associated with elevated ALT and AST levels. The association of a particular variable was expressed as odds ratio (OR) with a 95% confidence interval (CI). A two-tailed *P* value of.05 was considered statistically significant.

## RESULTS

The demographic, clinical and anthropometric characteristics of the participants have been displayed in Table 1. Among the 1014 patients enrolled in this study, 54.5% (n=553) were males and 45.5 % (n=461) were females. The mean age was 56.8+9.8 years. Seven hundred and thirteen patients (70.3%) were between the ages of 45 and 65 years and 445 (44.9%) patients had their diabetes diagnosed during the last five years. Three hundred and fifty patients (34.5%) were overweight and 572 (56.4%) were obese.

**Table 1:** The demographic, clinical and anthropometric characteristics of the 1014 patients with type 2 diabetes.

Variable	n (%)
Gender	
Male	553 (54.53)
Female	461 (45.46)

Age (years)	
25-45	146 (14.39)
46-65	713 (70.31)
> 65	155 (15.28)

Marital status	
Single	11 (1.1)
Married	974 (96.1)
Divorced	5 (0.5)
Widow	24 (2.4)

Body mass index (kg/m^2^)	
18.5-24.9	92 (9.07)
25-29.9	350 (34.51)
≥30	572 (56.41)

Diabetes duration (years)	
<5	455 (44.87)
5-10	271 (26.72)
11-15	125 (12.32)
>15	163 (16.07)

Waist circumference (cm)	
>102 (males)	282 (50.99)
>88 (females)	433 (93.92)

HBA_1c_ (%)	
>7	676 (66.66)

Lipids (mg/dL)	
LDL>100	498 (49.11)
TG>150	394 (38.85)
HDL<40 (males)	277 (50.09)
HDL<50 (females)	279 (60.52)

Liver enzymes U/L	
ALT>40	105 (10.35)
AST>38	55 (5.42)
ALT>40 and AST>38	46 (4.53)

Statin use	
Yes	642 (63.3)
No	372 (36.6)

Values are numbers of patients and percentage; total n=1014

Insulin was mostly prescribed for the old patients than for the younger group (*P*<.005). Hypertension was found to be most prevalent in patients who were over the age of 65 years (*P*<.005).

### Prevalence of elevated transaminases

**Figure 1:**
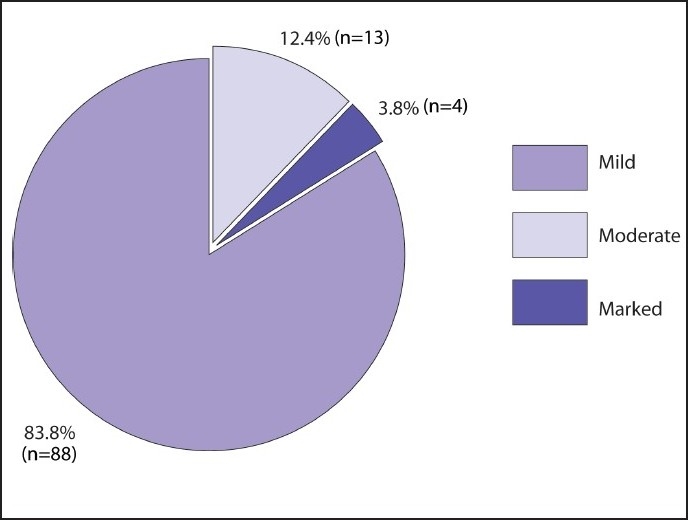
Degree of elevation of ALT enzyme level in patients with high readings. Mild, moderate and marked elevation indicate ALT levels 2 times, 2-3 times and more than 3 times the upper limit of normal value respectively.

Overall, the prevalence of elevated alanine transaminase (ALT) was 10.4%(n=105) with the gender-wise prevalence of 12.8% (n=71)(95% Confidence Interval (CI): 10.1, 15.5) in men, and 7.4% (n=34) (95% CI: 5.1, 9.7) in women (Table 2). The prevalence of elevated AST was 5.4% (n=56) (95% CI 4.1, 6.9) with the gender-wise prevalence of 5.6 %(n=31) (95%CI: 3.7, 7.5) in men and 5.4 % (n=25) (95% CI: 3.4, 7.5) in women). Only 4.5% (n=44) showed elevated levels of both ALT and AST. Of patients with high ALT levels, 88 patients (83.8%) had mild, 13 (12.4%) had moderate, and only four patients (3.8%) had marked elevation of the enzyme activity (Figure 1). The prevalence of elevated ALT and AST levels according to demographic, clinical, and anthropometric characteristics is shown in Table 2. Male gender (OR=2.35, CI: 1.5-3.8) and high waist circumference (OR=1.9, CI: 1.2-3.2) were associated with an increased risk of elevated ALT levels. Younger patients had a higher tendency to have elevated ALT compared to those over 65 years (OR=12.4 for patients aged 25-45 years and OR=5.8 for those who were 45-65 years old). Non-insulin use was associated with a high odds ratio for elevated ALT levels (OR=1.7, CI: 1.1-2.9). High ALT levels were significantly associated with the duration of diabetes: 14.3% (CI 11.2, 17.4), 9.2% (CI 5.9, 12.6), 8.0% (CI 3.4, 12.6), and 3.7% (CI 0.9, 6.4) in patients with diabetes dating to <5, 5-10, 11-15 and >15 years respectively. The prevalence of elevated AST level decreased with increased age and increased duration of diabetes (Table 2).

**Table 2:** Prevalence of elevated alanine transaminase and aspartate transaminase levels according to demographic, anthropometric and relevant characteristics.

Variable	Total	High ALT (%)				High AST (%)			
		n	Prevalence (95% CI)	χ^2^	*P* value	n	Prevalence (95% CI)	χ^2^	*P* value
Gender									
Male	553	71	12.8 (10.1, 15.5)	8.1	0.004	31	5.6 (3.7, 7.5)	0.02	.899
Female	461	34	7.4 (5.1, 9.7)			25	5.4 (3.4, 7.5)		

Age groups (years)									
25-45	146	29	19.9 (13.6, 26.1)	29.6	<0.005	12	8.2 (3.8, 12.7)	7.0	.031
46-65	713	73	10.2 (8.1, 12.4)			40	5.5 (3.9, 7.3)		
>65	155	3	1.9 (0.0, 4.0)			4	2.6 (0.1, 5.1)		

Smoking status									
Current	135	13	9.6 (4.8, 14.5)	0.11	0.947	8	5.9 (1.9, 9.9)	0.33	.849
Non	806	84	10.4 (8.4, 12.5)			45	5.6 (4.0, 7.2)		
Past	73	8	11.0 (3.8, 18.1)			3	4.1 (0.0, 8.7)		

Diabetes duration (year)									
<5	455	65	14.3 (11.2, 17.4)	15.7	0.001	37	8.1 (5.6, 10.6)	10.9	.012
6-10	271	25	9.2 (5.9, 12.6)			10	3.7 (0.9, 6.4)		
11-15	125	10	8.0 (3.4, 12.6)			4	3.2 (0.1, 6.3)		
>15	164	6	3.7 (1.4, 5.9)			5	3.1 (0.4, 5.7)		

Body Mass Index (kg/m^2^)									
18.5-24.9	92	9	9.8 (3.9, 15.7)	0.62	0.732	4	4.3 (0.2, 8.5)	0.93	.628
25-29.9	350	33	9.4 (6.5, 12.4)			17	4.9 (2.6, 7.1)		
>30	572	63	11.0 (8.5, 13.5)			35	6.1 (4.2, 8.1)		

Waist circumference(cm)									
Normal	299	27	9.0 (5.9, 12.2)	0.14	0.371	12	4.0 (1.8, 6.2)	1.4	.226
High	715	78	10.9 (8.7, 13.1)			44	6.2 (4.4, 7.9)		

Hemoglobin A1C (%)									
<7	338	34	10.1 (7.0, 13.2)	0.05	0.827	19	5.6 (3.2, 8.1)	0.01	.923
>7	676	71	10.5 (8.3, 12.7)			37	5.5 (3.8, 7.2)		

LDL (mg/dL)								
<100	516	48	9.3 (6.9, 11.7)	1.2	0.263	22	4.3 (2.5, 6.0)	3.2	.074
>100	498	57	11.4 (8.7, 14.2)			34	6.8 (4.6, 9.0)		

HDL (mg/dL)									
Normal	463	38	8.2 (5.8, 10.6)	1.4	0.040	20	4.3 (2.5, 6.2)	1.96	.162
Low	551	67	12.2 (9.5, 14.8)			36	6.5 (4.5, 8.6)		

Triglycerides (mg/dL)									
<150	620	60	9.7 (7.4, 11.9)	0.79	0.374	32	5.2 (3.4, 6.9)	0.40	.527
>150	394	45	11.4 (8.4, 14.5)			24	6.1 (3.7, 8.5)		

Hypertension									
No	239	35	14.6 (10.16, 19.13)	6.2	0.013	15	6.3 (3.2, 9.4)	0.33	.563
Yes	774	70	9.0 (7.02, 11.06)			41	5.3 (3.7, 6.9)		

Insulin use								
Yes	711	21	3.0 (1.7, 4.2)	5.4	0.019	14	4.6 (2.3, 7.0)	0.674	.412
No	303	84	27.7 (22.8, 32.6)			42	5.9 (4.2, 7.6)		

CI, Confidence interval, χ^2^, Chi-square test

### Multivariate analysis of risk factors for elevated ALT and AST

The only factors associated with elevated ALT levels in multivariate analysis were gender, waist circumference, age, and non- insulin use (Table 3). Male gender (OR=2.35, CI: 1.5-3.8) and higher waist circumference (OR=1.9, CI:1.2-3.2) were associated with increased prevalence of elevated ALT levels. Younger patients had a higher tendency to have elevated ALT levels compared to those over 65 years (OR=12.4 for patients between 25 and 45 years and OR=5.8 for those between 45 and 65 years). Non-insulin use was associated with high odds ratio for elevated ALT levels (OR=1.7, CI: 1.1-2.9). For elevated AST levels, only the age of 25-45 years was significantly associated with higher risk of elevated enzyme activity compared to age of more than 65 years.

**Table 3:** Multivariate analysis of factors associated with elevated ALT and AST*.

Variable	Odds ratio (95% CI)	*P* value
Gender		
Female	1	<.005
Male	2.4 (1.5-3.8)	

Age group (years)		
>65	1	
46-65	5.8 (1.8-18.7)	<.005
25-45	12.4 (3.7-41.9)	.003

Waist circumference (cm)		
Normal**	1	.008
High	1.9 (1.2-3.3)	

Insulin use		
Yes	1	.030
No	1.7 (1.1-2.9)	

* The only variable associated with elevated AST was age (OR=2.4, 95% CI: 1.05, 11.0)(25-45 year compared to >65 year)

** Normal values for men <102 cm, and <88cm for women.

## DISCUSSION

The prevalence of elevated transaminase levels is not known in the Jordanian population. In earlier studies, applying different methodology and enrolling variable population sample sizes as well as considering different cut-off values for ALT readings has yielded variable prevalence rates.^9^,^10^,^12^,^19^,^27^ The present study shows that ALT was elevated in 10.4% of our T2D patients (12.8% in males and 7.4% in females). Our results are consistent with the finding of Erbey et al. who reported prevalence rates of 10.7% and 5.3% in type 2 diabetic men and women respectively.^10^ Furthermore, West et al. reported a higher rate of 12.1%; 14.4% in men and 9.3% in women with T2D.^27^

**Figure 2:**
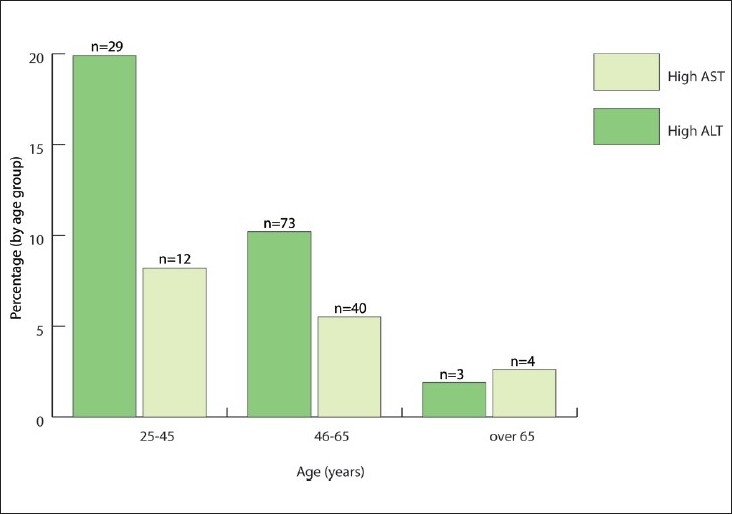
Pattern of ALT and AST elevation in the study population, according to different age groups. Note the higher prevalence of enzyme activity in the younger group with a decline as patients advance in age, and the relatively higher AST in the older group presumed to be a consequence of increased fibrosis.

This study showed that younger diabetic patients were more likely to have high ALT values than the older patients. However the older patients showed elevated AST activity (Figure 2). Supported by earlier studies,^2^,^7^,^28^ this finding suggested that severe steatosis denoted by a higher release of the ALT enzyme in response to hepatocytes derangement, tends to occur earlier in the disease process. As a marker of hepatocyte integrity the ALT activity decreases as steatosis progresses whereas inversely a rise in the AST level has been noticed in the older patients. The latter observation can be attributed to the fact that the clearance of this enzyme is mainly accomplished by the liver sinusoidal cells. While there is no effect from the necroinflammatory activity on AST level, advancing fibrosis which injures the sinusoidal cells leads to the relative increase in serum AST.^29^

The study concluded in multivariate analysis that both male gender and high waist circumference is independent predictor of ALT levels. We assume that the higher abdominal fat content in men compared to women would explains this finding which has been previously reported in other studies^27^,^30^,^31^

Body mass index was not associated significantly with elevated ALT levels. This finding can be attributed to the fact that elevated ALT levels are more related to central fat distribution than to general obesity. The most obvious explanation for this is that in the presence of insulin resistance, the larger mass of adipose tissue in abdominally obese subjects causes an inappropriate suppression of lipolysis and increased flux of non-esterified free fatty acid from visceral fat to liver. Hence, there is an increase in the size of hepatic FFA pool which favors the accumulation of triglycerides in the hepatocytes. The triglyceride overload provides abundant amounts of substrate for non-oxidative pathways, in addition to causing mitochondrial dysfunction. These lipid-induced changes are believed to ultimately lead to cell apoptosis.^32^,^33^

The findings of our study are similar to those of a study conducted by West et al, showing that insulin use may be protective against the effects of elevated ALT levels. Although this finding suggests that administration of insulin to diabetic patients may help to avoid harmful involvement of the liver by inflammation and fibrosis, it requires further investigation. This is because earlier studies have reported that the standardized mortality ratio from cirrhosis in diabetics was higher in patients who received insulin than in those treated by oral hypoglycemic agents.^34^

Our study has limitations. First, its cross-sectional design precludes the establishment of causal or temporal relations between T2D and elevated liver transaminase levels. Secondly, the performance of liver ultrasound examination was done only in patients with abnormal ALT levels rather than the entire patient sample. This probably makes our prevalence figure an underestimate as many earlier researchers have documented that fatty liver could be encountered even in people with normal ALT and AST levels.^33^,^35^,^36^

The prevalence of elevated ALT levels was 10.4% in our study population and ALT elevation was found to be more common in men than to women. There was also a significant association of elevated ALT levels with the duration of diabetes, lower HDL levels and the presence of fatty liver on ultrasound examination. Other risk factors for elevated ALT were younger age and larger waist circumference. Patients on insulin appeared to have lower ALT readings. These findings necessitate interference by lifestyle modification and early therapeutic measures to control risk factors, especially obesity, in younger diabetics which might help to prevent chronic liver disease.

## References

[1] Adami HO, Chow WH, Nyrén O, Berne C, Linet MS, Ekbom A (1996). Excess risk of primary liver cancer in patients with diabetes mellitus. J Natl Cancer Inst.

[2] Trombetta M, Spiazzi G, Zoppini G, Muggeo M (2005). Review article: Type 2 diabetes and chronic liver disease in the Verona diabetes study. Aliment Pharmacol Ther.

[3] Caldwell SH, Oelsner DH, Iezzoni JC, Hespenheide EE, Battle EH, Driscoll CJ (1999). Cryptogenic cirrhosis: Clinical charecterization and risk factors for underlying disease. Hepatology.

[4] Angulo P (2002). Nonalcoholic fatty liver disease. N Engl J Med.

[5] Marchesini G, Bugianesi E, Forlani G, Cerrelli F, Lenzi M, Manini R (2003). Non-alcoholic fatty liver, steatohepatitis, and the metabolic syndrome. Hepatology.

[6] Neuschwander-Tetri BA (2005). Non-alcoholic steato-hepatitis and the metabolic syndrome. Am J Med Sci.

[7] Coppack SW, Jensen MD, Miles JM (1994). In vivo regulation of lipolysis in humans. J Lipod Res.

[8] DeFronzo RA (1988). Lilly lecture 1987: The triumvirate: Beta-cell, muscle, liver. A collusion responsible for NIDDM. Diabetes.

[9] Salmela PI, Sotaniemi EA, Niemi M, Mäentausta O (1984). Liver function tests in diabetic patients. Diabetes Care.

[10] Erbey JR, Silberman C, Lydick E (2000). Prevalence of abnormal serum alanine aminotransferase levels in obese patients and patients with type2 diabetes. Am J Med.

[11] Clark JM, Brancati FL, Diehl AM (2003). The prevalence and etiology of elevated aminotransferase levels in the United States. Am J Gastroenterol.

[12] Kejariwal D, Phillips M, Dhatariya (2008). Abnormal liver function tests and diabetes mellitus: A secondary care prevalence study. Diabetes Med.

[13] Rosen F, Roberts NR, Nichol CA (1959). Glucocorticosteroids and transaminase activity: Increased activity of glutamic pyruvic transaminase in four conditions associated with gluconeogenesis. J Biol Chem.

[14] Westerbacka J, Cornér A, Tiikkainen M, Tamminen M, Vehkavaara S, Häkkinen AM (2004). Women and men have similar amounts of liver and intraabdominal fat, despite more subcutaneous fat in women: Implications of sex differences in markers of cardiovascular risks. Diabetologia.

[15] Kim HC, Jee SH, Han KH (2004). Normal serum aminotransferase concentration and risk of mortality from liver disease: Prospective cohort study. Br Med J.

[16] Ioannou GN, Weiss NS, Boyko EJ, Mozaffarian D, Lee SP (2006). Elevated serum alanine aminotransferase activity and calculated risk of coronary heart disease in the United States. Hepatology.

[17] DeRosa G, Swick RW (1975). Metabolic implications of the distribution of the alanine aminotransferase isoenzymes. J Biol Chem.

[18] Clark JM, Diehl AM (2003). Defining non-alcoholic fatty liver disease: Implications for epidemiological studies. Gastroenterology.

[19] Qari F, AlGhamdi A (2005). Fatty liver in overweight and obese patients in western part of Saudi Arabia: A study of sonological prevalence. Pak J Med Sci.

[20] Zelber-Sagi S, Nitzan-Kaluski D, Halpern Z, Oren R (2006). Prevalence of primary nonalcoholic fatty liver disease in a population based study and its association with biochemical and anthropometric measures. Liver Int.

[21] Daryani N, Mirmomen SH, Bahrami B, Nayerhabibi A (2003). Clinical and histological features of nonalcoholic steatohepatitis in Iranian patients. Acta Medica Iranica.

[22] American Diabetes Association (2008). Diagnosis and classification of diabetes mellitus. Diabetes Care.

[23] WHO (2000). Obesity: Preventing and managing the global epidemic. Report of a WHO Consultation. WHO Technical Report Series 894.

[24] (2001). Executive Summary of The Third Report of The National Cholesterol Education Program (NCEP) Expert Panel on Detection, Evaluation, And Treatment of High Blood Cholesterol In Adults (Adult Treatment Panel III): Expert Panel on Detection, Evaluation, and Treatment of High Blood Cholesterol in Adults. JAMA.

[25] Fraser CG, Cummings ST, Wilkinson SP, Neville RG, Knox JD, Ho O (1989). Biologic variability of 26 clinical chemistry analyses in elderly people. Clin Chem.

[26] Saadeh S, Younossi ZM, Remer EM, Gramlich T, Ong JP, Hurley M (2002). The utility of radiological imaging in nonalcoholic fatty liver disease. Gastroenterology.

[27] West J, Brousil J, Gazis A, Jackson L, Mansell P, Bennett A (2006). Elevated serum alanine transaminase in patients with type 1 or type 2 diabetes. Q J Med.

[28] Ioannou GN, Boyko EJ, Lee SP (2006). The prevalence and predictors of elevated serum aminotransferases activity in the United States in 1999-2002. Am J Gastroenterol.

[29] Kamimoto Y, Horiuchi S, Tanase S, Morino Y (1985). Plasma clearance of intravenously injected aspartate amminotransferase isoenzym: Evidence for preferential uptake by sinusoidal liver cells. Hepatology.

[30] Ruhl CE, Everhart JE (2003). Determinants of the association of overweight with elevated serum aminotransferase activity in the United States. Gastroenterology.

[31] Park HS, Han JH, Choi KM, Kim SM (2005). Relation between elevated serum alanine aminotransferase and metabolic syndrome in Korean adolescents. Am J Clin Nutr.

[32] Marceau P, Biron S, Hould FS, Marceau S, Simard S, Thung SN (1999). Liver pathology and the metabolic syndrome X in severe obesity. J Clin Endocrinol Metab.

[33] Kotronen A, Juurinen L, Hakkarainen A, Westerbacka J, Cornér A, Bergholm R (2008). Liver fat is increased in type 2 diabetic patients and underestimated by serum Alanine Aminotransferase compared with equally obese non-diabetic subjects. Diabetes Care.

[34] De Marco R, Locatelli F, Zoppini G, Verlato G, Bonora E, Muggeo M (1999). Cause-specific mortality in type 2 diabetes. The Verona Diabetes Study. Diabetes Care.

[35] Shimada M, Hashimoto E, Kaneda H, Noguchi S, Hayashi N (2002). Nonalcoholic steatohepatitis: risk factors for liver fibrosis. Hepatol Res.

[36] Vozarova B, Stefan N, Lindsay RS, Saremi A, Pratley RE, Bogardus C (2002). High alanine aminotransferase is associated with decreased hepatic insulin sensitivity and predicts the development of type 2 diabetes. Diabetes.

